# Screening and characterization of *Bacillus* sp. PE4 with polyethylene degradation capacity

**DOI:** 10.1128/spectrum.02533-25

**Published:** 2026-01-21

**Authors:** Yu-Hang Zhou, Zhong-Bao Jiang, Yue Fu, Zhen-Xing Tang, Hai Zhou, Jie Huang, Shang-Hong Xie, Hui-Yang Shen, Gao-Feng Bian, Lu-E Shi

**Affiliations:** 1 Department of Biotechnology, College of Life and Environmental Sciences, Hangzhou Normal University 26494, Hangzhou, Zhejiang, People's Republic of China; 2 School of Hospitality Management and Culinary Arts, Tourism College of Zhejiang 66363, Hangzhou, Zhejiang, People's Republic of China; 3 College of Material, Chemistry and Chemical Engineering, Key Laboratory of Organosilicon Chemistry and Material of Education, Hangzhou Normal University 26494, Hangzhou, Zhejiang, People's Republic of China; Instituto de Ecología, A.C. (INECOL), Pátzcuaro, Michoacán, Mexico

**Keywords:** polyethylene degradation, isolation, property, mechanism, pretreatment

## Abstract

Polyethylene (PE) is considered to be among the most extensively utilized plastics globally, and its extensive applications have led to significant environmental pollution. Therefore, finding a safe and efficient approach to degrade PE is crucial to mitigate its environmental impact. This study isolated five bacterial strains capable of degrading PE from agricultural soil covered with plastic mulch films. All strains were identified as *Bacillus* species, with *Bacillus* sp. PE4 demonstrating the highest degradation efficiency, as evidenced by a weight loss of 4.93% for PE films over 30 days. Scanning electron microscopy revealed cavities and cracks on the film surface after degradation by *Bacillus* sp. PE4. Culture conditions under pH 7.5, inoculum size 4.0%, and thermal pretreatment for PE samples significantly enhanced the degradation efficiency of *Bacillus* sp. PE4, and the weight loss rate reached up to 27.8% over 100 days, accompanied by a significant increase in extracellular peroxidase and polyphenol oxidase activities. This study established *Bacillus* sp. PE4 as an efficient PE-degrading bacterium, paving the way for investigating its degradation mechanisms and opening a new avenue for tackling PE pollution.

**IMPORTANCE:**

Polyethylene (PE) has been widely utilized in various fields, including packaging materials, agricultural films, and daily necessities. However, PE is not easily decomposed in the natural environment, leading to the accumulation of its residues. To find the efficient microbial degrading strains for PE, PE-degrading bacterial strains were isolated and screened from agricultural soil containing residual plastic mulch in Zhejiang province, China. The obtained *Bacillus* sp. PE4, one of the isolated PE-degrading strains, showed good degradation ability, and many approaches, including process optimization, were conducted to improve its degradation efficiency. The findings would provide valuable insights for screening novel PE-degrading microbial strains and developing more efficient microbial-based PE degradation strategies.

## INTRODUCTION

Polyethylene (PE), a widely used plastic material, is extensively produced and utilized globally because of its lightness, durability, and cost-effectiveness. It is commonly employed in various fields, including packaging materials, agricultural films, and daily necessities. However, PE’s characteristics, including high molecular weight, complex structure, three-dimensional morphology, strong physical and chemical stability, and hydrophobic surface, render it highly resistant to decomposition in the natural environment (1). Mulch film, one of the most common PE products, is primarily composed of light density PE (LDPE) and covers over 128,652 square kilometers of agricultural land worldwide (2). During its application and recycling processes, mulch film has been found to generate a substantial quantity of plastic residues. These PE residues not only alter the physicochemical characteristics of soil, including water-holding capacity, bulk density, and hydraulic conductivity, leading to reduced water retention and soil water shortage, but also adsorb heavy metal ions, toxic organic compounds, and antibiotics from the soil, thereby causing soil pollution (3–6). The contaminated soil indirectly affects plant nutrient uptake by influencing the richness and diversity of soil microorganisms, thus hindering plant growth (7, 8). Moreover, the presence of PE residues in the soil has been demonstrated to result in the ingestion of these by animals, which has been shown to lead to mortality among soil fauna and bioaccumulation through the food chain. This phenomenon has the potential to pose a threat to human health (9, 10).

The problem of PE residues in the soil has attracted widespread attention from researchers. PE degradation can be categorized into two types: abiotic and biotic. Abiotic degradation mainly includes photodegradation and thermal-oxygen degradation. At present, PE is mainly disposed of by landfill or incineration (11, 12). However, PE in landfills exhibits extremely slow degradation (5). The PE incineration process releases numerous harmful compounds, causing damage to the ecological environment. Therefore, it is particularly important to investigate the biodegradation approach of PE. The biodegradation of PE is mainly involved in insect chewing, the action of enzymes, and microbial degradation (13–18). Among these approaches, the degradation of PE by microorganisms has attracted considerable attention in recent years, with many studies emphasizing the capabilities of various microbial species and mechanisms to break down this ubiquitous plastic. Microbial degradation offers distinct advantages: (i) rapid microbial proliferation with low nutritional requirements (ii), cost-effectiveness, and (iii) suitability for large-scale production. These characteristics establish microbial degradation as a particularly sustainable and efficient strategy for PE waste management. It has been demonstrated that microorganisms, most notably bacteria and fungi, are capable of degrading PE by the formation of biofilms or hyphae on its surface. This process results in the formation of cracks and pores, thereby facilitating further permeation and degradation of PE (19–22). Many studies have demonstrated that fungi such as *Aspergillus oryzae* and *Mortierella subtilissima* and bacteria like *Bacillus*, *Rhodococcus*, and *Streptococcus* can effectively degrade PE by secreting enzymes that disrupt its polymer structure (23–27). At present, research has been concentrated on the optimization of microbial degradation processes and the exploration of new microbial strains with the capacity to degrade PE. Muangchinda et al. (28) observed the efficient degradation of LDPE using a bacterial consortium enriched from landfill sites, with dominant genera including *Mycobacterium*, *Cupriavidus*, and *Gordonia*. The findings suggested that microbial consortia, rather than individual strains, might offer a more effective approach to PE degradation. Putcha et al. (29) highlighted the potential of *Rhodococcus ruber* in degrading PE through the production of alkane hydroxylases and other enzymes. To improve the degradation of PE, many strategies on microbial degradation of PE have been explored. Research has demonstrated that pretreatment approaches such as UV irradiation and thermal oxidation can enhance the surface area and hydrophilicity of PE, thereby promoting microbial colonization and degradation (28).

The degrading efficiency of microorganisms is often limited by the complex and recalcitrant nature of PE, and further investigation is needed to find efficient microbial degrading strains and investigate the degradation process. Therefore, the study aimed to isolate PE-degrading bacterial strains from agricultural soil containing residual plastic mulch in Zhejiang province, China. The PE degradation capability of *Bacillus* sp. PE4, one of the isolated PE-degrading strains, was systematically evaluated using multiple analytical techniques, and many approaches, including process optimization, were conducted to improve degradation efficiency. Notably, this study represents the first comprehensive investigation examining the degradation of PE in different physical states (films versus powder) by the strains isolated in Zhejiang province, China. The findings would provide valuable insights for screening novel PE-degrading microbial strains and developing more efficient microbial-based PE degradation strategies.

## MATERIALS AND METHODS

### Screening and identification of degrading strains

#### PE samples and microbiological media

The PE samples used in this study consisted of a PE film (thickness is 0.05 mm) and PE powder (molecular weight is 100,000 Da and the density is 0.927 g/cm³) (Huachuang plastic Co. Ltd, China). PE films were cut into rectangular pieces and approximately weighed 0.020 g each and then sterilized for 15 min using a UV lamp. For PE powder, it was divided into 0.20 g each and also irradiated for 15 min using a UV lamp.

All PE samples were added into a basal liquid medium, with PE serving as the sole carbon source. The composition of the basal liquid medium was as follows: K₂HPO₄ 0.70 g/L, MgSO₄·7H₂O 0.70 g/L, KH₂PO₄ 0.70 g/L, NaCl 0.005 g/L, NH₄NO₃ 1.0 g/L, ZnSO₄·7H₂O 0.002 g/L, FeSO₄·7H₂O 0.002 g/L, and MnSO₄·H₂O 0.001 g/L. The basal solid medium required the addition of 18.0 g/L agar to basal liquid medium. The medium was sterilized at 121°C for 30 min.

#### The isolation of microorganisms degrading PE

Soil samples were collected in the autumn of 2023 from an abandoned farmland to the north of Hangzhou Normal University, where visibly degraded PE residues (e.g., with aging, surface cracks, or pores) were present. The samples were stored in sterile bags and promptly transported to the lab for immediate processing. About 10.0 g of obtained samples was initially co-incubated with preweighed PE powders in 100 mL of the basal liquid medium at 28°C, with agitation at 180 rpm for 40 days. Afterward, the incubated samples were passaged into a fresh basal liquid medium for 40 days, with PE powders serving as the sole carbon source.

At the termination of the culture, the samples were then serially diluted and plated onto Luria-Bertani agar medium to isolate individual colonies. Each isolated strain was subsequently inoculated into a basal solid medium with PE powders as the sole carbon source and incubated at 28°C to screen for PE-degrading capability.

#### Identification of the microbial bacterium

Molecular identification of PE-degrading isolates was performed through 16S rRNA gene sequencing with the degrading capability of PE. The following primers were utilized for polymerase chain reaction (PCR) amplification of 16S rDNA: 27F (5′-AGA GTT TGA TCC TGG CTC AG- 3′) and 1492R (5′-GGTTACCTTGTTACGACTT-3′). The cycling conditions were as follows: 1 cycle of 2 min at 95°C, followed by 30 cycles of 1 min at 94°C, 1 min at 55°C, and 2 min at 72°C, and 1 final cycle of 5 min at 72°C. The presence of PCR products was detected using agarose gel electrophoresis, and the qualified PCR products were sent to Sangon Biotech Co. Ltd (Shanghai, China) for sequencing. The sequencing results were then analyzed using a software called Bioedit v7.7.1.0 (Tom Hall, Vista, CA, USA), and the sequences with a high level of confidence were recorded. A comparison was made between the intercepted sequences and the sequences in the NCBI-BLAST database. The strains were identified based on the similarity of their sequences with the alignment results.

### Analysis of PE degradation characteristics

#### Growth determination of the strains

To assess the growth ability of the obtained PE-degrading strains (PE 1–5), each isolate was inoculated at 2.0% (vol/vol) into a basal liquid medium containing PE as the sole carbon source, with an uninoculated medium serving as the control under identical cultivation conditions. The culture was then incubated at 28°C, with agitation at 180 rpm, over a duration of 30 days. After the cultivation, 3.0 mL of fermentation broth was transferred into a glass cuvette, and the OD_600_ nm of each sample was measured using a spectrophotometer (Crystal Technology & Industries Inc., USA).

#### Determination of dry weight of PE film residues

Strains PE 1–5 were inoculated at 2.0% (vol/vol) into a basal liquid medium, with the PE film serving as the sole carbon source. The culture was then incubated at 28°C, with agitation at 180 rpm, over a duration of 30 days. The basal liquid medium without the strain was used as a control. At the end of cultivation, the PE film was washed using 2.0% (wt/vol) sodium dodecyl sulfate for 4 h and then dried at 50°C overnight. Finally, the weightlessness rate A% was calculated according to the formula A% = (M_0_-M_1_) /M_0_ × 100%, where M_0_ is the initial weight of PE film (g) and M_1_ is the residual weight of PE film (g).

#### Determination of PE crystallinity

Strains PE 1–5 were inoculated at 2.0% (vol/vol) into the basal liquid medium with PE powder as the sole carbon source. The culture was incubated at 28°C with shaking at 180 rpm for 30 days. The basal liquid medium without the strain was used as a control. After 30 days of incubation, PE powder was washed and dried with the method following “*Determination of dry weight of PE film residues.”* The crystallinity of PE powder was determined by isodensities. Briefly, PE powder was added into 20 mL of anhydrous ethanol contained in a sealed centrifuge tube. Sterile water was then added dropwise and thoroughly mixed with PE powder. PE powder was suspended in the middle of the solution and remained suspended for 1 min. The volume (V) and weight (M) of the solution were measured, respectively. The density (ρ) of PE powder could be calculated using the following formula: ρ= M/V. The crystallinity (X_c_) of PE powder was calculated using the formula: Xc= (ρ-ρ_a_）/ (ρ_c_-ρ_a_）, where X_c_ is the degree of crystallinity, ρ is the measured density of the sample, ρ_a_ is the density of completely amorphous PE (0.854 g/cm³), and ρ_c_ is the density of fully crystalline PE (1.014 g/cm³).

#### Analysis of enzyme activity

After the incubation of the strains as described in the section of “*Determination of dry weight of PE film residues,”* the fermentation broth was subjected to centrifugation at 10,000 rpm for 10 min to obtain a cell-free supernatant. For polyphenol oxidase (PPO) activity assay, the supernatant (1.0 mL) was mixed with catechol solution (1.5 mL) and 2.5 mL of phosphate buffer (pH 7.0). The OD_525 nm_ of the mixed solution was measured. PPO activity was calculated using the formula: U/L = (ΔOD_525_)/(0.01 × T × 0.001), where U is the enzyme activity unit, which is defined as the amount of enzyme required to achieve a change in ΔOD_525_ of 0.01 per min, T is reaction time, and 0.001 is the conversion factor for unit standardization.

For peroxidase (POD) activity assay, 2.6 mL of phosphate buffer (0.10 mol/L) was mixed with 0.10 mL of o-phenylenediamine-ethanol solution, 0.20 mL of hydrogen peroxide solution (0.30%), and 0.10 mL of the supernatant. The OD_430 nm_ of the mixed solution was measured. POD activity was calculated using the formula: U/L = (ΔOD_430_)/(0.01 × T × 0.001), where U is the enzyme activity unit, which is defined as the amount of enzyme required to achieve a change in ΔOD_430_ of 0.01 per min), T is reaction time, and 0.001 is the conversion factor for unit standardization.

#### Analysis of surface morphology of the PE film

The PE film was treated following the method “*Determination of dry weight of PE film residues.”* After treatment, the PE film was visually observed, and photographs were taken. Subsequently, the PE film was immobilized on the sample stage using a conductive adhesive, sprayed with gold at 6 mA for 1 min, and finally the surface topography of PE film was analyzed by scanning electron microscopy (SEM) (30).

#### Analysis of functional group of PE samples

The changes in the structure of PE samples after being treated by PE-degrading strains were measured by Fourier transform infrared coupled attenuated total reflectance spectroscopy (Thermo Nicolet IS 20) in the frequency range from 4,000 to 400 cm^−1^ (31).

#### Analysis of nontargeted metabolites

PE samples were treated with PE-degrading strains following the method of “*Determination of dry weight of PE film residues.”* The fermentation broth after the degrading treatment was for nontargeted metabolome analysis, and the untreated basal medium was used as the control. UPLC-Q-Exactive Plus MS technology was used to perform full-scan analysis of the samples in the data-dependent acquisition mode. Chromatographic separation was carried out using a UPLC system (Nexera X2 LC-30AD, Shimadzu, Japan) equipped with a chromatography column (ACQUITY UPLC HSS T3, 2.1 × 100 mm, 1.8 μm; Waters, USA). Mass spectrometric analysis was performed using a mass spectrometer (Q Exactive Plus, Thermo Scientific, USA). Peak extraction and metabolite identification were carried out by a software MSDIAL version 4.9, and multidimensional statistical analysis of mass spectrometry data was performed using Python.

### Investigation of PE degradation process

#### Effect of mixed strain and mutagenic strain fermentation on PE degradation efficiency

Single colonies of each strain grown on the plates were inoculated into Luria-Bertani liquid medium. The cultures were then subjected to agitation at 180 rpm, 37°C for 24 h to obtain the bacterial suspension. The suspension underwent centrifugation at 10,000 rpm for 10 min to obtain the bacterial cells. The obtained cells were then re-suspended in an equal volume of sterile normal saline, thus preparing the bacterial suspension.

The optimization of the degrading strain was divided into two parts: mixed strain fermentation and mutagenic strain culture. Three co-culture combinations (PE 1+ PE 4, PE 3+ PE 4, and PE 1+ PE 3+ PE 4) were employed. The mutagenesis procedure was involved in exposing the strain suspension (OD_600_ ≈ 0.60 in sterile saline) to UV irradiation (254 nm, 30 W) for 50 s at a distance of 30 cm. Immediately following UV treatment, the cells were protected from light for 1 h to prevent photoreactivation. Strain PE 4, used as the control in this study, was cultured under identical conditions. The strain (mixed strain, mutagenic strain, and the control strain PE4) was inoculated into a liquid medium, with the PE film serving as the sole carbon source following the method of “*Determination of dry weight of PE film residues.”* The cultures were incubated at 28°C with agitation at 180 rpm for 100 days. During the incubation period, to maintain nutrient availability, a fresh basal medium was added every 14 days. After the end of the culture, the dry weight of PE film residues and enzyme activity were determined, respectively.

#### Effect of pH and inoculum size on PE degradation efficiency

The strain was inoculated into the basal liquid medium, with the PE film serving as the sole carbon source. Effects of pH values (5.5, 6.5, 7.5, 8.5, 9.5, and 10.5) on PE degradation efficiency were evaluated. An inoculum size of 2.0% was used. Effects of inoculum size (2.0%, 4.0%, 6.0%, 8.0%, and 10%) on PE degradation efficiency were studied. The pH was maintained at the initial pH of the basal medium, while other conditions remained unchanged. The basal liquid medium without the strain was used as the control. The cultures were incubated at 28°C with agitation at 180 rpm for 100 days. During the incubation period, a fresh basal medium was added every 14 days to maintain nutrient availability. Residual PE film dry weight and enzyme activity were determined, respectively.

#### Pretreatment for PE films

The effect of different PE pretreatment approaches on the degradation efficiency of PE films was evaluated using residual PE film dry weight and enzyme activity as indicators. The pretreatment approaches for PE films included the following: (i) water bath at 100°C for 60 min (ii), UV irradiation for 60 min, and (iii) concentrated nitric acid treatment for 60 min. After pretreatment, PE films were washed three times using sterile water, then air-dried, and finally added into the culture medium as the sole carbon source. The control group, inoculated with strain PE4 and supplemented with the untreated PE film, was included under otherwise identical conditions. All cultures were initiated at pH 7.0 with a 2.0% inoculum size and incubated at 28°C for 100 days with agitation at 180 rpm. To maintain nutrient availability, a fresh basal medium was added every 14 days. After incubation, the residual dry weight of the PE film and enzyme activity were determined, respectively.

### Statistical analysis

Each experiment was repeated in triplicate. Statistical analysis was performed using software Origin Pro 2021 and IBM SPSS Statistics. Student’s *t*-test (*P* < 0.05) was used to determine significant differences between the data.

## RESULTS

### Isolation and identification of PE-degrading bacteria

**Fig 1:**
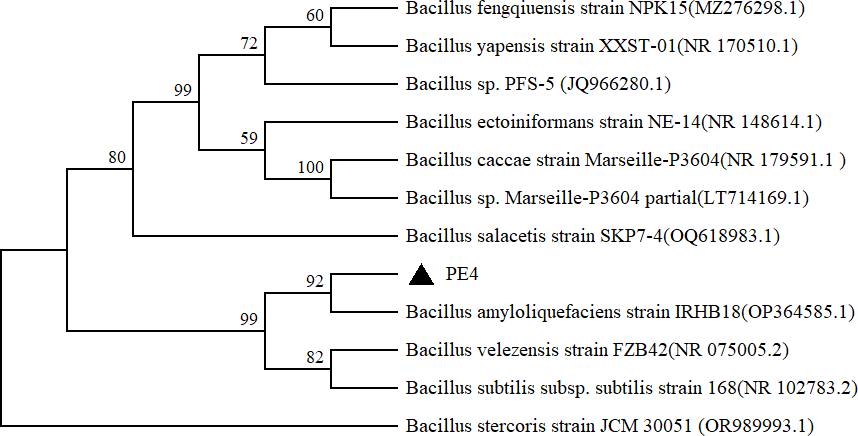
Phylogenetic tree of *Bacillus* sp. PE4 based on 16S rDNA gene sequence analysis.

In the present study, five strains, named PE 1–5, were obtained using PE powders as the sole carbon source. According to the 16S rRNA gene sequences of the five strains, it was found that all of the isolates belonged to *Bacillu*s sp. Specifically, a phylogenetic analysis clearly positioned strain PE4 within *Bacillu*s sp. (Fig. 1). Furthermore, BLASTN analysis against the NCBI database also confirmed that the 16S rDNA sequence of PE4 shared a high similarity of (99%) with the strains *Bacillu*s sp. (Table 1).

**TABLE 1:** Sequence similarity analysis of the 16S rDNA gene from *Bacillus sp*.^*a*^

Strain	Blast NCBI	Identities	GenBank accession
*Bacillus sp*. PE4	*Bacillus amyloliquefaciens* IRHB18	1447/1450 (99%)	OP364585.1
	*Bacillus amyloliquefaciens* BS22	1442/1445 (99%)	KR063202.1
	*Bacillus velezensis* strain HN_2	1443/1444 (99%)	MK310268.1
	*Bacillus velezensis* strain A68	1444/1447 (99%)	PQ813729.1
	*Bacillus subtilis* strain RA206	1440/1443 (99%)	KJ564127.1
	*Bacillus subtilis* strain 102	1436/1437 (99%)	OR673305.1

^*a*^ PE4 performed with BLASTN algorithm via the NCBI database.

### Analysis of PE degradation characteristics

#### Growth determination of the strains

**Fig 2:**
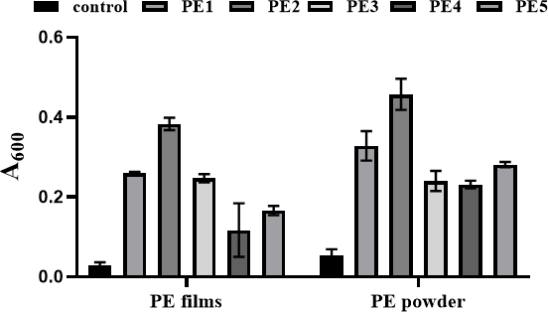
OD_600nm_ of the fermentation broth with different PE samples after 30 days (the control: basal liquid medium with PE serving as the sole carbon source without bacterial inoculation).

After incubating the five bacterial strains in a basal liquid medium with PE samples as the sole carbon source with an inoculum size of 2.0% (vol/vol), at 28°C and 180 rpm for 30 days, OD₆₀₀ values of the fermentation broth were measured. The results revealed that all PE-degrading strains exhibited better growth ability when utilizing PE powder as the sole carbon source, in comparison to the PE film (Fig. 2). Among the five bacterial strains tested, *Bacillus* sp. PE2 and *Bacillus* sp. PE4 showed the highest OD values and the lowest OD values at 600 nm, respectively (Fig. 2).

#### Determination of dry weight of PE film residues

**Fig 3:**
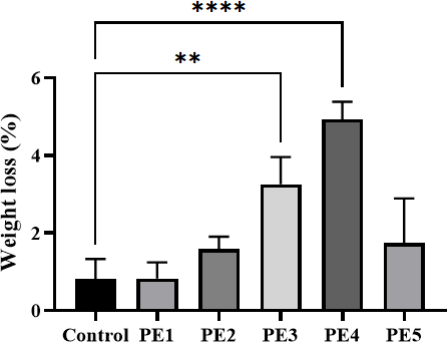
Weight loss rate of the PE film after 30 days of degradation by isolated strains (the control: basal liquid medium with the PE film serving as the sole carbon source without bacterial inoculation) **P < 0.01, ****P < 0.0001.

To evaluate PE degradation efficiency, the weight loss of films incubated with individual strains for 30 days was compared (Fig. 3). Among the tested strains, *Bacillus* sp. PE4 exhibited the highest degradation efficiency, with a weight loss of 4.93%, whereas *Bacillus* sp. PE1 showed the lowest degradation efficiency (0.83%); no obvious weight loss was detected in the control.

#### Determination of PE crystallinity

**Fig 4:**
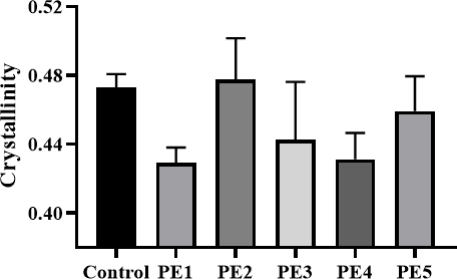
Crystallinity of PE powders after 30 days of degradation by different strains (the control: untreated PE powders).

After 30 days of cultivation using PE powder as the sole carbon source, the crystallinity of PE powder was determined by the isodensity method. As shown in Fig. 4, the crystallinity of PE powder degraded by *Bacillus* sp. PE4 was the lowest (0.43) compared to that of the control group, while the sample treated with *Bacillus* sp. PE2 showed the highest crystallinity (0.48).

#### Analysis of the surface morphology of the PE film

**Fig 5:**
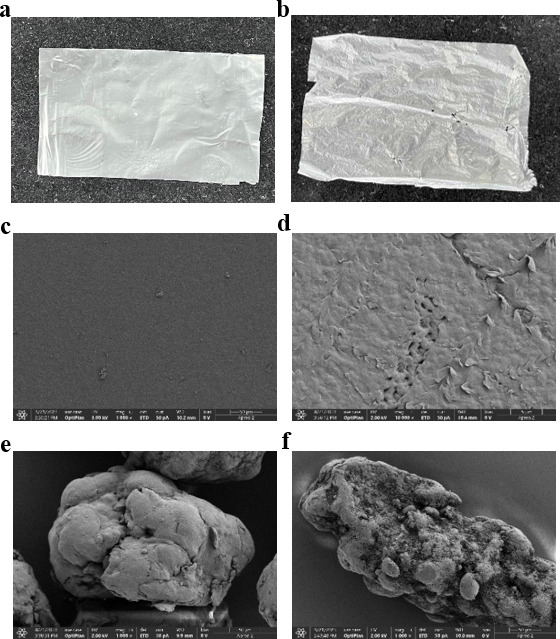
The surface morphology of PE before and after degradation by *Bacillus* sp. PE4 after 30 days. (**a**) Visual appearance of the PE film before degradation. (**b**) Visual appearance of the PE film after degradation. (**c**) SEM of PE Films before degradation. (**d**) SEM of PE Films after degradation. (**e**) SEM of PE powders before degradation. (**f**) SEM of PE powders after degradation.

After 30 days of culture, PE film samples were visually observed and analyzed by SEM. As shown in Fig. 5, PE samples’ surface exhibited distinct morphological changes after the degrading treatment. The control groups maintained intact surfaces with smooth textures with no detectable cracks or voids (Fig. 5a, c and e). After being treated by *Bacillus* sp. PE4, PE samples showed significant surface modifications, including shrinkage deformation, increased grooves in PE powder, and severe damage to the PE film manifested as pits, cracks, and cavities (Fig. 5b, d and f).

#### Analysis of functional group of PE films

**Fig 6:**
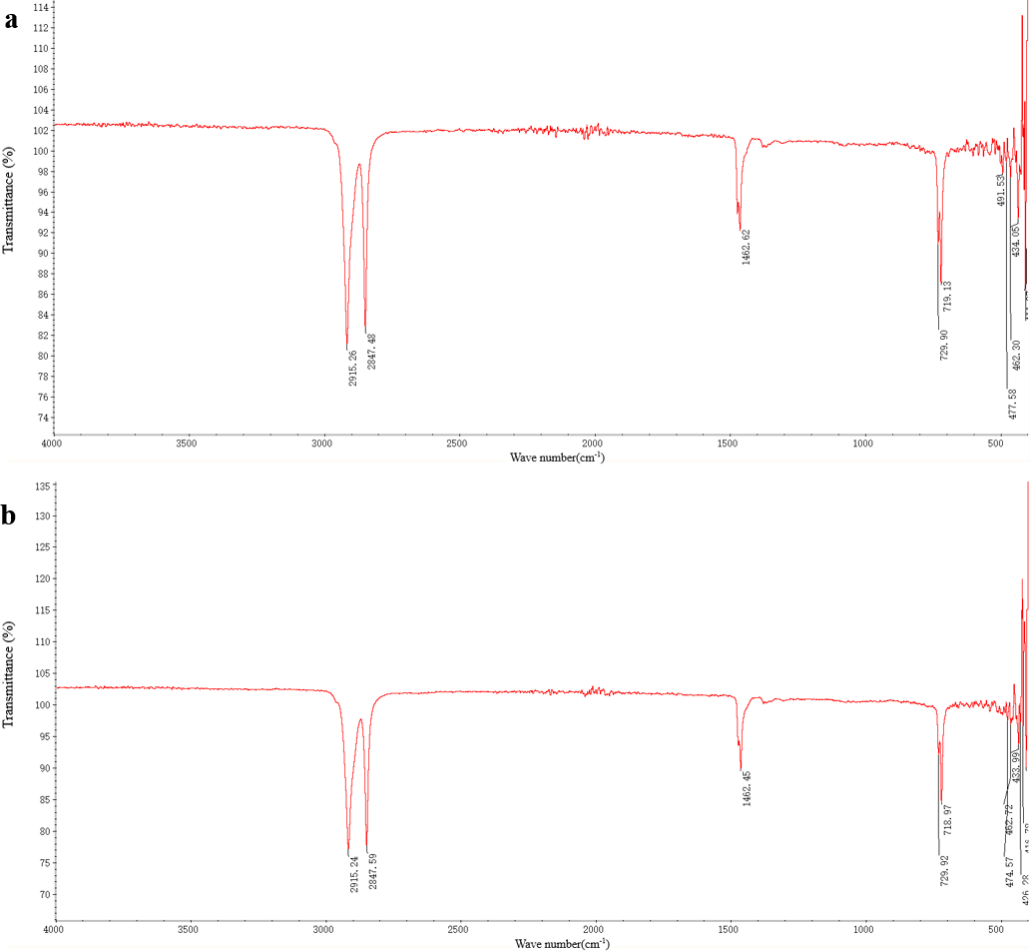
Fourier transform infrared spectra of PE films before and after degradation by *Bacillus* sp. PE4 after 100 days. (**a**) Untreated PE film. (**b**) Treated PE film.

The functional group changes of the PE film were analyzed by Fourier transform infrared spectroscopy (FTIR). The results showed that the control had characteristic vibration peaks at 718 cm⁻¹ (backbone motions), 1,462 cm⁻¹ (C-H bending), and 2,847/2,914 cm⁻¹ (CH₂ stretching), with the degraded film showing reduced transmittance indicative of structural alterations (Fig. 6a). The film in the treatment group still had characteristic peaks at the same wave number, but the transmittance was reduced, and no new oxidized functional group (e.g.*,* carbonyl, C=C bond) characteristic peaks were detected (Fig. 6b).

#### Analysis of nontargeted metabolites

**Fig 7:**
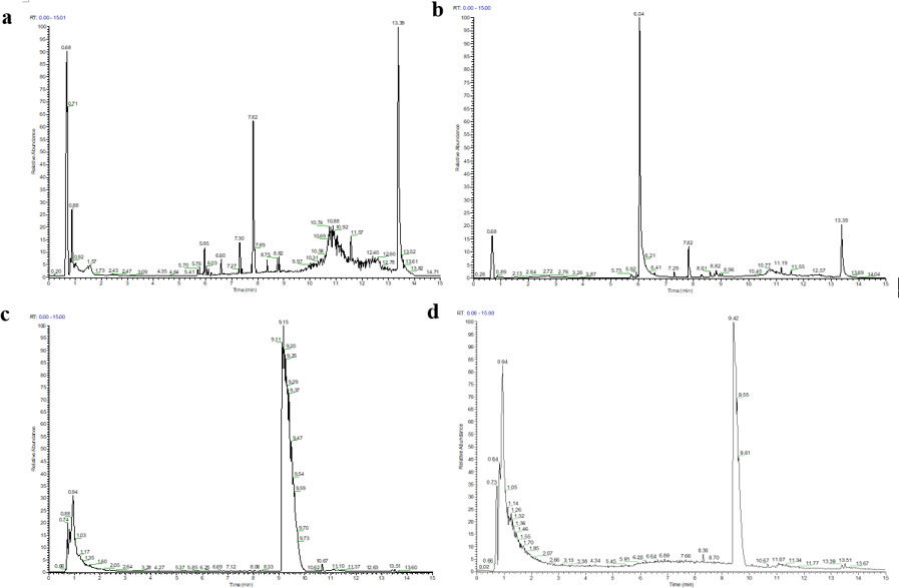
LC-MS/MS mass spectrometry of PE films before and after degradation by *Bacillus* sp. PE4 after 100 days. (**a**) Positive-ion mass spectrum of the PE film before degradation. (**b**) Positive-ion mass spectrum of the PE film after degradation. (**c**) Negative-ion mass spectrum of the PE film before degradation. (**d**) Negative-ion mass spectrum of the PE film after degradation.

Nontargeted metabolomic analysis of the fermentation broth was performed using UPLC-Q-Exactive Plus MS. The results revealed that, compared with the control group (Fig. 7a and c), the *Bacillus* sp. PE4 treatment group exhibited new characteristic peaks at retention time points of 6.04 min in the positive ion mode and 0.94 min in the negative ion mode (Fig. 7b and d). Detected metabolites included short-chain carboxylic acids (e.g., octanoic acid), dicarboxylic acids (e.g., adipic acid), and aldehydes (e.g., decanediol).

### Investigation of the PE degradation process

**Fig 8:**
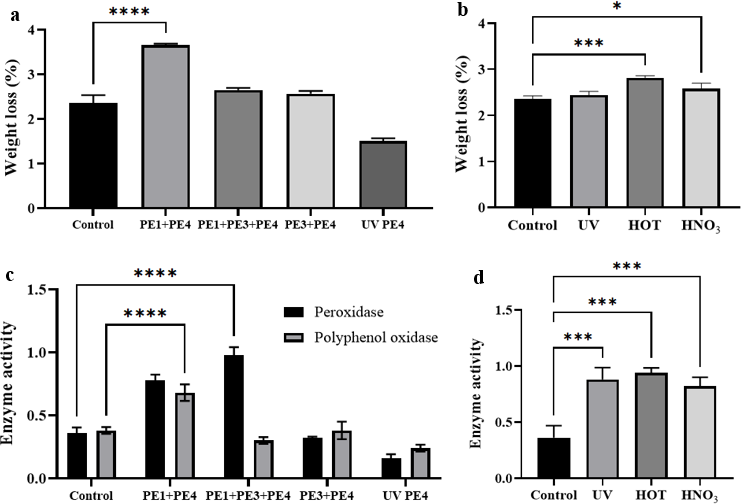
Weight loss rate and extracellular enzyme activity of the PE film after 100 days of degradation. (**a**) Weight loss rate of the PE film under different strains (co-cultures, mutagenized strains, and the pure strain PE4 as a control). (**b**) Weight loss rate of the PE film after pretreatment of PE (UV: UV irradiation for 60 min; HOT: water bath at 100°C for 60 min; HNO₃: nitric acid treatment for 60 min, with untreated PE film as a control). (**c**) Extracellular enzyme activity after degradation. (**d**) Extracellular enzyme activity after pretreatment of PE *P < 0.05, ***P < 0.001; ****P < 0.0001.

Three mixed-strain combinations (PE1+PE4, PE3+PE4, and PE1+PE3+PE4) were established, and UV mutagenesis of PE4 was performed (254 nm, 30 W, distance 30 cm, irradiation for 50 s, followed by 1 h dark incubation). Using *Bacillus* sp. PE4 as the control, the weight loss rate of PE film was measured after 100 days. The results showed that the PE1+PE4 co-culture group achieved the highest degradation efficiency, with a weight loss rate increased by 155% compared to the control, while the UV-mutated PE4 group showed only 65.8% of the control’s efficiency (Fig. 8a).

In pH gradient experiments (5.5, 6.5, 7.5, 8.5, 9.5, and 10.5), *Bacillus* sp. PE4 exhibited optimal degradation efficiency at pH 7.5, with a film weight loss rate of 27.8%, compared to 8.5% in the control. Inoculum size gradient tests (2.0%, 4.0%, 6.0%, 8.0%, and 10.0%) revealed that 4.0% inoculum size yielded the best degradation efficiency, showing a film weight loss rate of 23.4% versus 8.5% in the control.

Three pretreatment methods (100°C water bath for 60 min, 254 nm UV irradiation for 60 min, and concentrated nitric acid immersion for 60 min) were evaluated using the untreated PE film as the control. After 100 days, the thermal pretreatment group demonstrated the highest degradation enhancement with a 120% increase in the weight loss rate compared to the control, followed by UV pretreatment (105% increase). The nitric acid treatment group showed a lower enhancement than the thermal pretreatment group (Fig. 8b).

### Enzyme activity assay

The cell-free supernatant was obtained by centrifuging the fermentation broth from each experimental group (10,000 rpm, 10 min), and the activities of PPO and POD were measured. The results demonstrated that both enzyme activities positively correlated with PE degradation efficiency during the degradation process by *Bacillus* sp. PE4, with POD activity exhibiting significantly greater variation than PPO activity (Fig. 8c and d). The mixed-strain group PE1+PE4 and water bath (100°C) pretreatment group showed higher activities of both enzymes compared to other experimental groups, while the UV-mutated PE4 group displayed lower enzyme activities than those of the pure PE4 control group.

## DISCUSSION

### Screening and identification of PE-degrading strains

At present, many studies have revealed that the strains from *Bacillus* sp. are capable of degrading PE, which are consistent with the isolation of five *Bacillus* strains (PE1–PE5) from agricultural soil containing residual PE films in the present study (Fig. 1, Table 1). Using a dual selective enrichment approach with PE powder as the sole carbon source, Bitalac et al. (32) successfully isolated bacterial strains belonging to 10 different genera, including *Bacillus* sp. In the study of Park and Kim (33), a variety of bacteria from the sediments of municipal landfills were isolated. The most effective species of bacteria in terms of degradation was *Bacillus* sp., which was able to reduce the dry weight of PE particles by 14.7% after 60 days, as well as the average particle size by 22.8% after the same amount of time. Sixty species of marine bacteria from pelagic waters were isolated by Kumar and Jha (34). The authors determined their ability to degrade LDPE. The results showed that three of these strains were positive for growth in the PE sample as the sole carbon source, and the isolates were identified as follows: *Kocuria palustris* M16, *Bacillus pumilus* M27, and *Bacillus subtilis* H1584. These findings were in agreement with our results, indicating that *Bacillus* sp. showed potential ability to degrade PE.

### PE degradation capability of *Bacillus* sp. PE4

In this study, all PE-degrading strains showed a preference for PE powder as a substrate (Fig. 2). This preference was likely due to the physical properties of PE powder. Its smaller particle size provided a larger specific surface area, which offered increased microbial colonization and enhanced the enzyme accessibility to the polymers, thus improving degradation efficiency. Felgel-Farnholz et al. (35) validated this hypothesis by analyzing the degradation behavior of different PE types during mechanical recycling. Their findings indicated that smaller PE particles degraded more readily due to their increased surface area.

Among the five *Bacillus* strains, *Bacillus* sp. PE2 exhibited the strongest growth ability (highest OD₆₀₀), while *Bacillus* sp. PE4 showed the weakest growth (Fig. 2). Notably, despite its weak growth, *Bacillus* sp. PE4 exhibited the highest degradation efficiency. It exhibited the highest PE film weight loss (4.93%) and the most significant reduction in PE powder crystallinity (Fig. 3 and 4) after 30 days. The lower PE crystallinity, suggestive of a lower melting point and a more loosely packed molecular structure, enhanced the polymer’s accessibility to microbes. This correlation explained the high degradation efficiency of *Bacillus* sp. PE4 against both the PE film and powder. It was worth noting that the observed discrepancy between limited microbial growth and efficient PE degradation suggested a potential trade-off in resource allocation. Under certain conditions, bacteria may prioritize synthesizing and secreting extracellular degradative enzymes (e.g., esterases and oxidases) over rapid biomass accumulation, particularly when faced with a recalcitrant primary carbon source like PE. Thus, this “slow growth, high activity” phenotype aligned with the classic physiological trade-off between microbial growth and investment in adaptive traits such as substrate adaptation and stress response (36). Specifically, as exemplified in *B. subtilis*, the strain could achieve enhanced fitness in specific functions by reallocating proteomic resources from biosynthetic pathways to specialized adaptive pathways, a strategy often mediated by global regulators (37). This principle might explain why *Bacillus* sp. PE4, despite its slower growth, demonstrated superior PE degradation capability. This phenomenon was not unique to *Bacillus* sp. PE4. Zhuang et al. (38) reported significant differences in LDPE degradation efficiency among *Bacillus* sp., noting that *B. subtilis* ATCC6051 could form biofilms on the untreated LDPE film, whereas other *Bacillus* strains lacked this ability. Yoon et al. (39) observed that *B. cereus* had relatively weak growth on the PE film but still achieved effective PE degradation. The authors attributed it to the secretion of unique lipolytic enzymes by *B. cereus*, which accelerated PE oxidation and decomposition. Similarly, Tribedi and Sil (40) found that certain *Pseudomonas* spp. showed limited growth in carbon-limited media but secreted higher levels of extracellular enzymes (e.g., esterases and oxidases) to degrade LDPE.

The morphological damage observed on PE (e.g., pits, cracks, and cavities on films and increased grooves on powders) provided clear evidence that the degradation by *Bacillus* sp. PE4 resulted in physical damage commonly observed in the degradation of PE samples (Fig. 5). These observations agreed with previous findings by Nedi et al. (41), who isolated PE-degrading microorganisms from a landfill in Adama, Ethiopia, and documented crack formation on degraded PE surfaces through SEM. Bitalac et al. (15) also found a similar observation to ours. The authors isolated 10 LDPE-degrading strains from marine sediments in Manila Bay. After 60 days of co-cultivation with LDPE, these strains were observed growing within surface fissures, creating honeycomb-like pits and increasing the surface roughness.

Beyond surface morphological changes, FTIR analysis provided further evidence of degradation by revealing distinct chemical alterations in PE after exposure to *Bacillus* sp. PE4 (Fig. 6). Despite many studies reporting oxidative degradation characteristics, such as new carbonyl groups in *Pseudomonas pseudofirmus* MQ-1-treated PE or C=C bonds in LDPE degraded by coastal bacteria (34, 42), no significant oxidative changes were observed in our study (Fig. 6). These results suggested that *Bacillus* sp. PE4 might employ non-oxidative degradation mechanisms, potentially involving enzymatic chain scission without oxygen incorporation, as proposed by Danso et al. (43). *Bacillus* sp. PE4 degraded PE through potentially non-oxidative pathways, which needs to be assessed with further mechanistic investigation.

To elucidate the molecular degradation pathway of PE by *Bacillus* sp. PE4, nontargeted metabolomic analysis via UPLC-Q-Exactive Plus MS was performed (Fig. 7). This approach identified key degradation metabolites, providing direct evidence for the breakdown of polymers into small molecules. As one of the olefinic polymers, PE consists of repeating ethylene units ([–CH_2_–CH_2_–]_n_) generated through catalytic polymerization. Similar to paraffin and long-chain alkanes, PE constitutes the simplest structural form of high-molecular-weight alkane polymers (44). Compared to the control group, new characteristic peaks exhibited a retention time of 6.04 min in positive ion mode and 0.94 min in negative ion mode mass spectrometry, indicating the formation of novel degradation metabolites at these time points (Fig. 7). These findings were in accordance with those of Costa et al. (45), who reported that PE degradation metabolites exhibited polarity-dependent retention. In our study, the peak at 6.04 min corresponded to polar short-chain/dicarboxylic acids, which showed stronger retention, whereas the earlier eluting peak at 0.94 min aligned with moderately polar aldehydes/alcohols (Fig. 7). This confirmed that these peaks were attributed to PE breakdown rather than intrinsic microbial metabolism. Numerous recognized biomarkers of PE degradation compounds, including short-chain carboxylic acids (e.g., 2-methylpropanoic acid), dicarboxylic acids (e.g., adipic acid), and phthalaldehyde (e.g., O-phthalaldehyde), have been detected (42, 46, 47). The metabolites including short-chain carboxylic acids (e.g., octanoic acid), dicarboxylic acids (e.g., adipic acid), and aldehydes (e.g., decanediol) in degraded PE films were also observed in our results (Fig. 7). Our results were consistent with previously reported patterns of microbial PE degradation. Costa et al. (45) identified aliphatic dicarboxylic acids as PE oxidative degradation signatures, while Ghatge et al. (48) and Yao et al. (49) confirmed short-chain carboxylic acids (C₂–C₁₆) and aldehydes as terminal PE cleavage products. LC-MS identified definitive biomarkers for PE degradation by *Bacillus* sp. PE4 (Fig. 7). The detection of adipic acid, for example, indicated dual-site oxidation and C-C scission of polymer backbone, a key intermediate step. These results collectively indicated that such compounds could serve as reliable biomarkers for PE film degradation.

To improve the PE-degrading efficiency of *Bacillus* sp. PE4, three optimization approaches, namely, microbial community interactions (e.g., mixed cultures and mutagenesis), environmental factors (e.g., pH and inoculum size), and PE pretreatment, were investigated. In a microbial community, each strain can execute metabolic pathways independently or through cooperation among different strains. As shown in Fig. 8a, the PE1+PE4 co-culture demonstrated the highest degradation efficiency (155%) compared to the control group, supporting the hypothesis that mixed cultures generally showed higher PE degradation compared to the single culture. The results were consistent with those of the work of Wang et al. (50), who found that *Rhodococcus* sp. RS and *B*. aryabhattai 5-3 as optimal consortium for mulch film degradation. In addition to co-culture, UV-mediated mutagenesis of the strain represented another viable strategy to improve PE degradation efficiency. UV mutagenesis has been widely employed to improve bacterial strains’ traits. Dong et al. (51) and Ning et al. (52) enhanced the performance of ammonia-oxidizing bacteria and thermophilic bacteria, respectively, for sludge hydrolysis through UV mutagenesis. However, UV-mutated *Bacillus* sp. PE4 showed reduced performance (65.8% of the control), indicating a suboptimal outcome under our experimental conditions (Fig. 8a and b). While targeted mutagenesis has been successfully employed to enhance PE degradation, its efficacy is known to depend heavily on methodological and strain-specific factors. Future work should therefore focus on optimizing mutagenesis protocols, such as by employing more precise genetic engineering techniques, to improve the success rate for PE-degrading strains.

Effects of pH and inoculum size of *Bacillus* sp. PE4 on PE degradation were investigated. The results demonstrated that *Bacillus* sp. PE4 exhibited the highest degradation efficiency at pH 7.5, with a PE film weight loss of 27.8%, compared to only 8.5% in the control group (results not shown). The effect of pHs on PE degradation efficiency has been reported in many investigations (41, 53). pH not only affects bacterial growth but also influences extracellular degrading enzymes secreted by the strains. Nedi et al. (41) revealed that pH 7.2 was optimal for the growth of most PE-degrading bacteria, where both bacterial growth and PE degradation performance were favorable. These results were consistent with our results. However, in the study of Zhang et al. (53), the authors found that the multicopper oxidase exhibited the highest enzymatic activity under acidic conditions (pH 4.5), significantly enhancing PE degradation. In our study, the highest degradation efficiency was observed at an inoculum size of 4.0%, where PE film weight loss reached 23.4%, compared to only 8.5% in the control (results not shown). Inoculum size also plays a critical role in the degradation of PE as it directly influences microbial growth and degradation efficiency. Mallisetty et al. (54) found that increasing the inoculum size from 1.0% to 4.0% significantly improved the growth and degradation performance of *Paenibacillus* sp. and *Serratia* sp. for LDPE, with the highest efficiency at 4.0%. However, further increases led to a decline in both growth performance and degradation ability for PE samples. The results were consistent with our findings. El-Shanshoury (55) also reported that an inoculum size of 4.0% was optimal for the biodegradation of LDPE by a marine bacterial consortium composed of *B. licheniformis*, *B. subtilis*, and *B. mojavensis*. Besides pH and inoculum size, other factors including temperature and PE content influenced the PE degradation efficiency. Mouafo et al. (56) reported that temperature significantly affected the PE degradation efficiency of *P. aeruginosa*, with the highest degradation rate at 44°C. Nedi et al. (41) indicated that *P. aeruginosa* PDI-1 and *P. balearica* PDI-17 exhibited the highest growth performance at a PE concentration of 0.50%. These findings provided valuable insights for the future optimization of *Bacillus* sp. PE4 for enhanced PE degradation.

PE pretreatment is an efficient approach for improving PE degradation efficiency. PE pretreatment disrupts the polymer’s surface structure, thereby enhancing its susceptibility to microbial biodegradation. In our present work, three methods of PE pretreatment were investigated. As shown in Fig. 8b, pretreated PE groups exhibited significantly higher degradation efficiency than the control, with thermally treated samples demonstrating the greatest enhancement (120%). The results indicated that heat-treated PE samples were more readily degraded. Maleki Rad et al. (57) reported that thermo-oxidative pretreatment enhanced the biodegradation of LDPE by *Achromobacter denitrificans* Ebl13, as evidenced by the increased degradation rate and more pronounced surface alterations. This finding agreed with the results of our present work (Fig. 8b). UV is one of the popular methods for PE pretreatment (20, 58). UV irradiation enhances the surface roughness of PE, thereby promoting microbial colonization and subsequent degradation by providing more attachment sites. However, in our present study, UV pretreatment only produced a moderate improvement for PE degradation (105%), possibly due to suboptimal irradiation time or insufficient UV intensity (Fig. 8b). Nitric acid treatment served to oxidize PE chains, introduced polar functional groups, and reduced the molecular weight, all of which contributed to enhanced biodegradability. Chaudhary et al. (59) demonstrated that LDPE pretreated with nitric acid showed improved degradation by *Cephalosporium* species. FTIR analysis revealed the formation of hydroxyl and carbonyl groups, and SEM observations showed significant surface morphological changes.

The biodegradation of PE is initiated by microbial extracellular enzymes that cleave the chemical bonds in its otherwise recalcitrant polymer chains. In *Bacillus* sp. PE4, the activities of two key enzymes (PPO and POD), which showed a significant positive correlation with degradation efficiency, were critical for PE biodegradation (60). Given that POD activity showed more pronounced changes than PPO during the degradation process (Fig. 8c and d), it was proposed to be the primary enzyme responsible for PE breakdown in *Bacillus* sp. PE4. In contrast, PPO likely played a secondary role, such as metabolizing aromatic intermediates from PE scission or modifying the PE surface to facilitate enzyme access. The highly active enzyme activity profile was particularly notable given the strain’s weak growth (Fig. 2 to 5). *Bacillus* sp. PE4 achieved the highest PE degradation efficiency yet yielded the lowest biomass among the tested strains. Furthermore, the absence of new oxidative functional groups (e.g., carbonyls, C=C bonds) in Fourier transform infrared spectra of degraded PE (Fig. 6) indicated that overt surface oxidation was not a primary degradation pathway. Collectively, these observations demonstrated that secreted enzymes, rather than biomass accumulation or surface oxidation, constituted the principal mechanism for efficient PE degradation by *Bacillus* sp. PE4 (Fig. 8). Enzymatic strategies for the PE degradation strategy have been well-established in many investigations. Mukherjee and Kundu (61), for example, reported that manganese peroxidase and lignin peroxidase—enzymes functionally analogous to POD characterized in this study—could effectively degrade PE, with synergistic interactions between them further enhancing degradation efficiency. Although PPO and POD were the focus of our study, it is noteworthy that other enzymes, most prominently laccase, have also been widely reported to facilitate PE degradation (62). Fujisawa et al. (63) demonstrated a laccase-mediated system could degrade PE films via oxidative scission, whereas Santo et al. (59) identified a thermostable PE-degrading laccase from *Rhodococcus ruber* C208 that maintained optimal activity at 70°C. Collectively, these studies revealed a diverse range of enzymes capable of degrading PE, underscoring the need to identify and characterize the entire enzyme systems of *Bacillus* sp. PE4. In summary, the strong correlation between PPO/POD activity and PE degradation, along with the lack of oxidative surface modifications, confirmed that extracellular enzymes were central to the degradation mechanism of *Bacillus* sp. PE4. Our findings provided an initial mechanistic explanation for the “weak growth but high degradation” trait and establish a solid foundation for future research. Subsequent work should focus on purifying and characterizing *Bacillus* sp. PE4-derived PPO/POD, exploring potential synergies between these enzymes, and identifying other degrading enzymes (e.g., laccases) that may contribute to the PE-degrading capacity.

### Conclusion

In this study, five strains degrading PE samples were screened. Among them, a bacterium, *Bacillus* sp. PE4, showed the best degradation ability for PE samples. *Bacillus* sp. PE4 could colonize PE samples, leading to a reduction in both relative crystallinity and molecular weight, along with the formation of surface crevices. The structural alterations observed in PE samples demonstrated the degradation capability of *Bacillus* sp. PE4, as evidenced by a 4.93% weight loss of PE over 30 d. Key enzymes, including PPO and POD, were involved in the degradation process. Culture pH and inoculum size significantly affected the PE degradation efficiency. The degradation efficiency was significantly influenced by culture pH and inoculum size, with the optimal performance achieved at pH 7.5 and a 4.0% inoculum. Furthermore, a co-culture of strains PE1 and PE4 led to enhanced degradation. In short, this study indicates that *Bacillus* sp. PE4 is an effective PE-degrading strain, offering a promising microbial solution for addressing PE pollution.

## Data Availability

The 16S rDNA gene sequence of *Bacillus* sp. PE4 was deposited in the NCBI 16S rRNA GenBank database (BLASTN) under accession number PX518828. Data will be made available on request.
